# Water-Supply

**Published:** 1911-04-15

**Authors:** A. Saxon Snell


### INSTITUTIONAL LETTER-BOX.

### WATER-SUPPLY.

*To the Editor of* The Hospital.

Sir,—The writer of your special institutional article in this week's issue of The Hospital says "the allowance of water for all purposes per person per diem may be reckoned at from twenty to thirty gallons." I should be interested to know of any modern hospital which finds this allowance sufficient. My experience is that they use from fifty to seventy gallons per diem.—Yours, etc.,

A. Saxon Snell.

[The amount of water required by an institution depends, of course, entirely upon its special needs, and all that one can do is to give a general average. That given (20-30 gallons per head per diem for all purposes) is, we admit, below the average given by the large London hospitals (35 gallons per day), but most of these institutions consume large quantities of water in their laboratories and medical schools. The average of ten Continental institutions (with medical schools) works out at thirty-two gallons per head; that of two hospitals with large hydropathic departments, at eighty-six gallons, that of eight Continental and American hospitals without schools at twenty-eight gallons. In any case provision has to be made for a larger quantity of water than is actually consumed. The figures given by our correspondent seem too high an average to be compatible with strict economy.—Ed. The Hospital.]

